# Occurrence of *Taenia* species and *Toxoplasma gondii* in pigs slaughtered in Bujumbura city, Kayanza and Ngozi provinces, Burundi

**DOI:** 10.1186/s12917-024-04445-6

**Published:** 2024-12-31

**Authors:** Salvator Minani, Emma Spiessens, Alyssa Labarrière, Pascal Niyokwizera, Anastasie Gasogo, Jean-Bosco Ntirandekura, Chiara Trevisan, Sarah Gabriël

**Affiliations:** 1 Department of Biology, Faculty of Sciences, University of Burundi, Bujumbura, Burundi; 2 Department of Public Health, Institute of Tropical Medicine, Antwerp, Belgium; 3 Laboratory of Foodborne Parasitic Zoonoses, Department of Translational Physiology, Infectiology and Public Health, Faculty of Veterinary Medicine, Ghent University, Merelbeke, Belgium; 4 National Veterinary Laboratory, Directorate of Animal Health, Ministry of Environment, Agriculture and Livestock, Bujumbura, Burundi; 5 Department of Animal Health and Productions, Faculty of Agronomy and Bio-Engineering, University of Burundi, Bujumbura, Burundi

**Keywords:** Occurrence, *Taenia solium*, *Taenia hydatigena*, *Toxoplasma gondii*, Pigs, Burundi

## Abstract

**Background:**

*Taenia* spp. and *Toxoplasma gondii* are foodborne parasites affecting humans and pigs. The magnitude of the burden of these parasites in pigs in Burundi is not known. Therefore, this study aimed to estimate the prevalence of *Taenia* spp. infections in pigs by meat inspection, partial carcass dissection and molecular confirmation and estimate the prevalence of *Toxoplasma gondii* infection in pigs by serology. A cross-sectional study was conducted in pig slaughter slabs located in Bujumbura city, Kayanza and Ngozi provinces. Multisampling strategies were used to sample 576 pigs. Upon pig slaughter, blood samples were collected to perform indirect ELISA for detecting antibodies against the *T. gondii* P30 protein in the serum. Routine meat inspection was carried out to detect *T. solium* and *T. hydatigena* cysticerci. The tongue, heart and masseter muscles were dissected by making slices less than 5 mm thick to estimate the intensity and stages of *T. solium* cysticerci. A subset of cysticerci and suspected lesions per infected pig were examined using PCR-RFLP to differentiate *Taenia* spp.

**Results:**

Of the 576 pigs, 14 (2.4%) were positive for *T. solium* cysticercosis by meat inspection and 67 (11.6%) by partial carcass dissection. After molecular analysis, 66 (11.5%) samples were confirmed to be *T. solium* infections. The average of *T. solium* cysticerci in the dissected organs was estimated at 80 cysticerci. Most cysticerci (76.1%) were counted in the masseter muscles, followed by the tongue (18.8%) and the heart (5.1%). The majority of cysticerci (88.3%) were viable, 6.4% were calcified and 5.3% were degenerated. Approximately 69% of the pigs infected with *T. solium* had light infections, 13.4% had moderate infections and 17.9% had heavy infections. Thirty-two out of 576 pigs (5.5%) were suspected of being infected with *T. hydatigena* by meat inspection, but 24 pigs (4.2%) were confirmed molecularly to be positive for *T. hydatigena* infection. The seroprevalence of *T. gondii* infection in pigs was 17.7%.

**Conclusions:**

This study indicates that *T. solium* and *T. gondii* parasites are endemic in Burundi and provides evidence of potential public health risks for the local population. Effective control strategies, including improved pig farming practices, better hygiene and sanitation, increased meat inspection, monitoring of infected pigs, risk-free culinary practices, and treatment of tapeworm carriers, should be implemented to avoid the perpetual contamination of pigs and humans with these zoonotic parasites.

## Background

*Taenia* spp. and *Toxoplasma gondii* are among the foodborne parasites contributing to a high societal and health burden [1]. *Taenia solium* is a zoonotic parasite with a complex two-host life cycle, including humans as definitive hosts, and pigs and accidentally humans as intermediate hosts [2]. It is endemic in low- and middle-income countries, including those in sub-Saharan Africa, South and Central America, Southeast Asia, and the Western Pacific [1]. Humans acquire *T. solium* taeniasis by eating raw or undercooked pork containing metacestode larval stages (cysticerci), which results in the development of an adult pork tapeworm in the small intestine [2]. Most patients with pork tapeworms remain asymptomatic, though they may experience abdominal pain, diarrhoea, bloating, and nausea [2]. Humans can get accidentally infected by ingesting fruits, vegetables, and water contaminated with eggs from adult tapeworm (*T. solium*) carriers, leading to (neuro)cysticercosis, a major cause of acquired epilepsy in sub-Saharan Africa [3, 4]. Patients with neurocysticercosis may develop symptoms several years after infection, including epilepsy, hydrocephalus, severe headaches, stroke, and dementia, with epilepsy being the most common neurological disorder [5]. Pigs develop *T. solium* cysticercosis by ingesting human faeces, feed, and water contaminated with eggs from adult tapeworms, resulting in the development of cysticerci in muscles and organs [6]. Pigs infected with *T. solium* typically do not show clinical signs. Still, in rare cases, they may develop myositis in locomotion, somnolence, chewing disorders, seizures in heavily infected pigs, and loss of consciousness [7, 8]. Pigs can also get infected with *Taenia hydatigena*, a non-zoonotic cestode whose metacestode larval stages develop in the organs and membranes of the thoracic and abdominal cavities, while dogs and other canids act as definitive hosts, harbouring the adult tapeworm in their small intestines [9]. *Toxoplasma gondii* is a cosmopolitan zoonotic parasite that infects all warm-blooded species, including pigs and humans as intermediate hosts, and cats and other felids as definitive hosts [10]. Humans acquire toxoplasmosis through ingestion of undercooked meat containing *T. gondii* cysts and oocysts from water, soil, or food contaminated with cat faeces [11]. They can get infected through transplacental transmission, blood transfusion or organ transplantation, and accidental inoculation of tachyzoites [11]. Most cases of acquired human toxoplasmosis are asymptomatic, but congenital toxoplasmosis can cause abortion, stillbirth, or result in newborns developing neurological and ocular disorders such as encephalitis, hydrocephalus, chorioretinitis, intracranial calcifications, and mental retardation [12, 13]. Pigs, like other livestock and wild animals, become infected through ingestion of oocysts from environmental contamination with cat faeces; ingestion of cysts in the tissues of infected birds, rodents, or cannibalism, and by vertical transmission [10]. *Toxoplasma gondii* infection in pigs is asymptomatic, but can lead to abortions during pregnancy [14].

Epidemiological studies on *T. solium* and *T. gondii* infections have been conducted in many countries around the world and have shown a high prevalence of these infections in Africa [15–18]. Higher prevalences for *T. solium* and *T. gondii* infections were usually observed in pigs and humans in countries with inadequate hygiene and sanitation, traditional pig farming practices, and inadequate meat inspection [1, 10]. In Burundi, pig farming is a major source of income for resource-poor farmers, where risk factors for *Taenia* spp. and *T. gondii* transmission are abundant [19]. A prevalence of 15.5% by tongue palpation and 31.5% by antibody enzyme-linked immunosorbent assay (Ab-ELISA) was reported for *T. solium* cysticercosis in pigs and humans, respectively [19, 20]. The prevalence of toxoplasmosis was 44.1% by Ab-ELISA and indirect immunofluorescence test for humans, whereas no data is currently available for toxoplasmosis in pigs [21]. Although some epidemiological data on *T. solium* cysticercosis are available in Burundi, updated data using more optimal diagnostic techniques are needed. Full carcass dissection with meat slices less than 5 mm thick is considered the gold standard diagnostic technique for estimating *T. solium* occurrence, cysticerci stages and infection intensity levels in endemic areas [22]. Partial carcass dissection including only the heart, tongue and masseter muscles showed satisfactory performance results, with a sensitivity estimated at 81% [23]. Considering the labour-intensive process and high costs of purchasing pigs for full carcass dissection, researchers are recommended to use partial carcass dissection to save time, labour, and costs [22, 23]. A study unravelling the occurrence and burden of *Taenia* spp. and *T. gondii* in pigs is needed to provide evidence-based results and to advocate for the development and implementation of control measures against these parasites in the future. Therefore, this study aimed to (i) estimate the prevalence of *Taenia* spp. infections based on meat inspection, partial carcass dissection, and molecular confirmation and (ii) estimate the prevalence of *T. gondii* infection in pigs using serology.

## Results

### Pig slaughter slab facilities and sample description

**Fig. 1:**
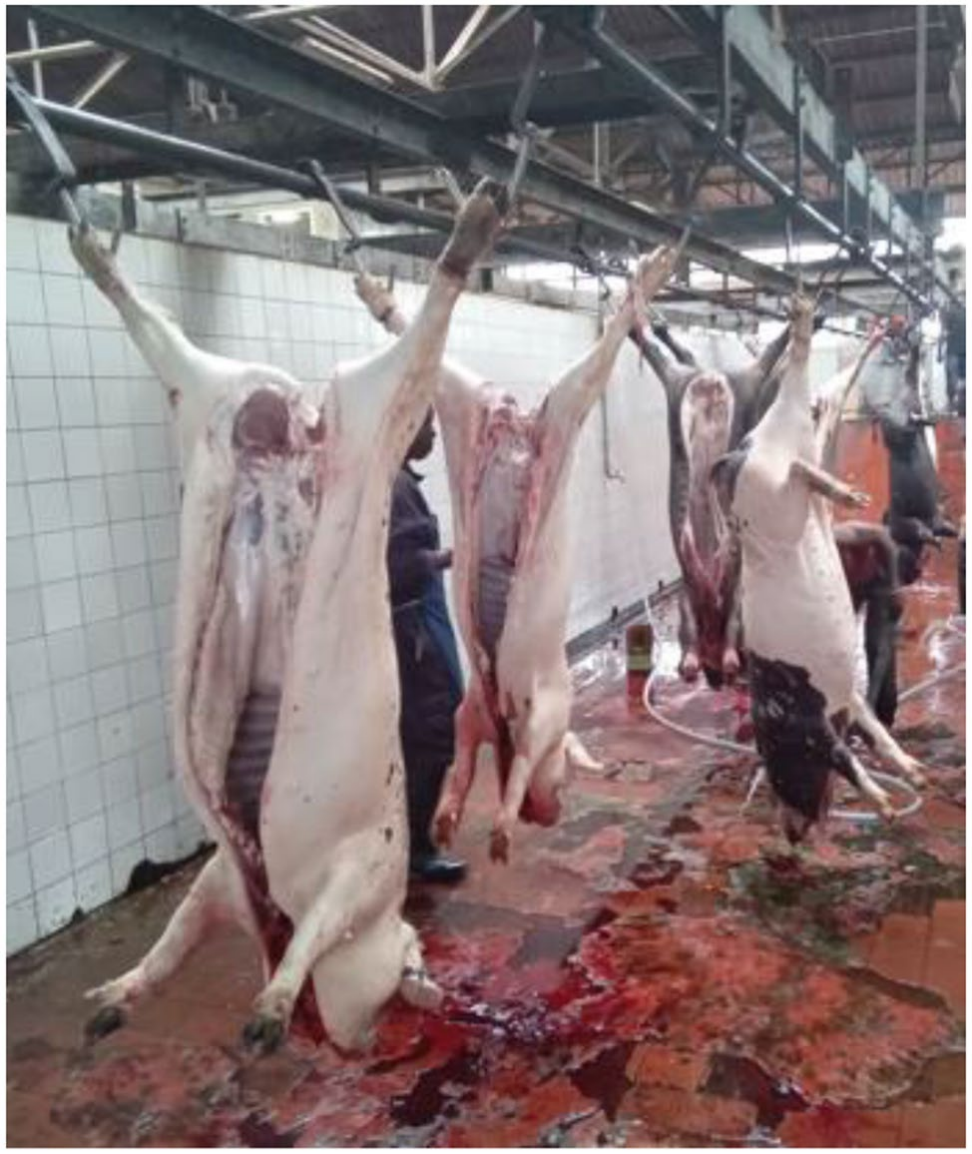
Pigs slaughtered at the national slaughterhouse in Bujumbura city

**Fig. 2:**
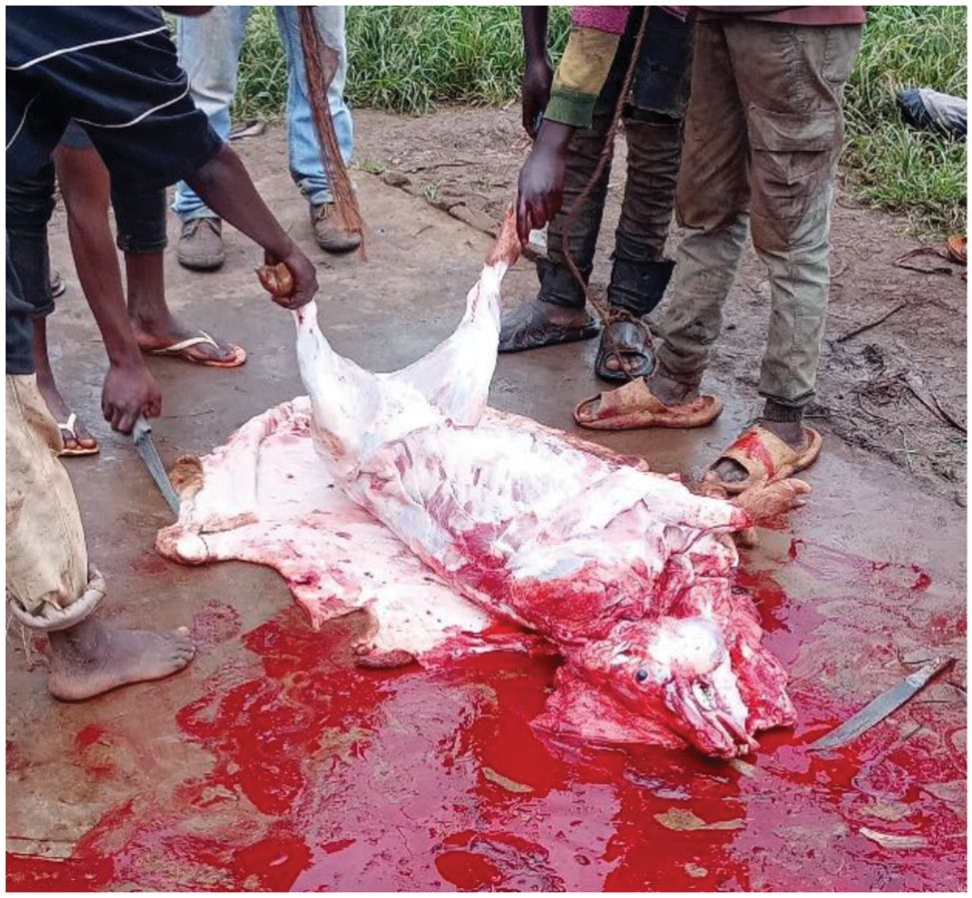
An example of a pig slaughter slab in the study area (Gikoma, Kayanza and Ngozi)

Of all the pig slaughter slabs visited, only the national slaughterhouse in Bujumbura city adhered to basic standards including appropriate facilities and hygienic standards (Fig. 1). In contrast, other pig slaughter slabs, such as in Gikoma in Bujumbura city and those in Kayanza and Ngozi provinces, had low levels of hygiene and cleanliness, with pigs being slaughtered outdoors on wooden or cemented floors, or even in the bush (Fig. 2). A total of 576 pigs were sampled in different slaughter slabs. More than half of the pigs (320/576, 55.6%) were females. The age of the pigs ranged from 6 months to 36 months with an average age of 14 months. Sixty percent of the pigs (60%) were cross breeds (large white), while 40% were local breeds (black pigs).

### Prevalence of *Taenia* spp. infections using meat inspection, partial carcass dissection and molecular confirmation

**Fig. 3:**
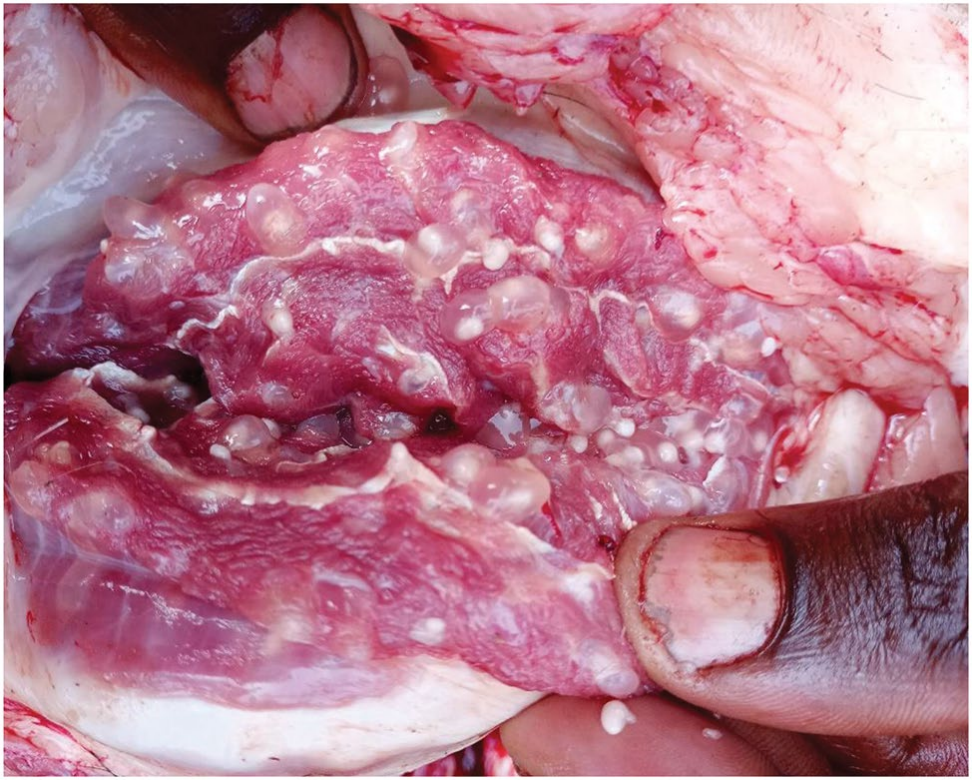
An example of a masseter muscle heavily infected with *Taenia solium* cysticerci

**Fig. 4:**
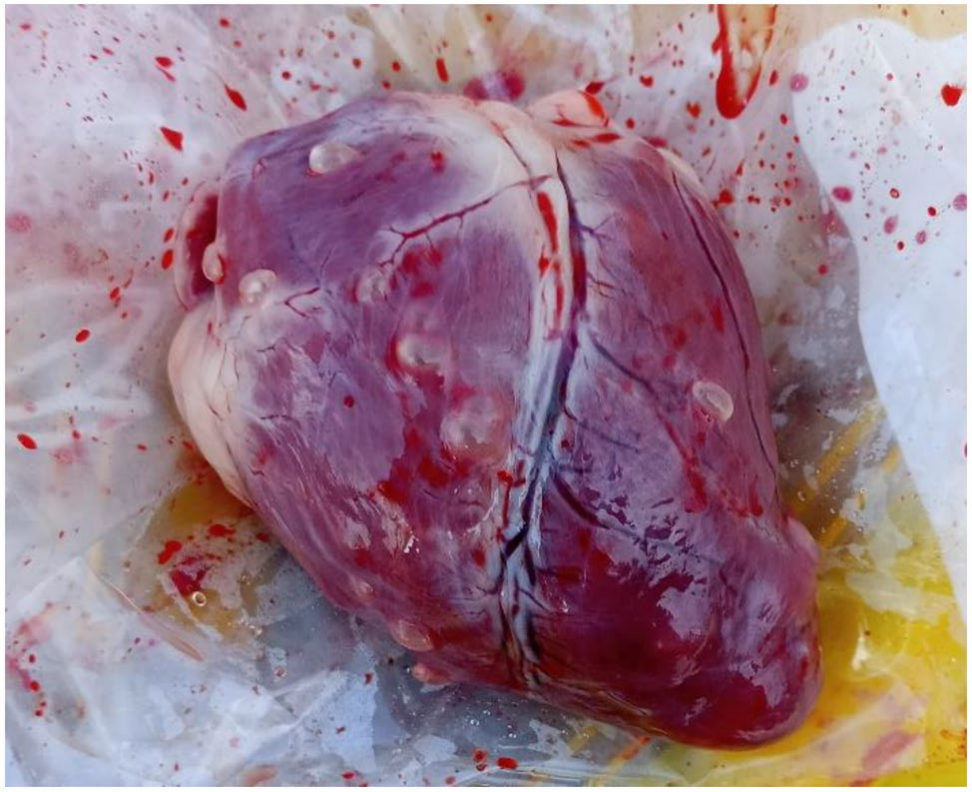
An example of a heart heavily infected with *Taenia solium* cysticerci

**Fig. 5:**
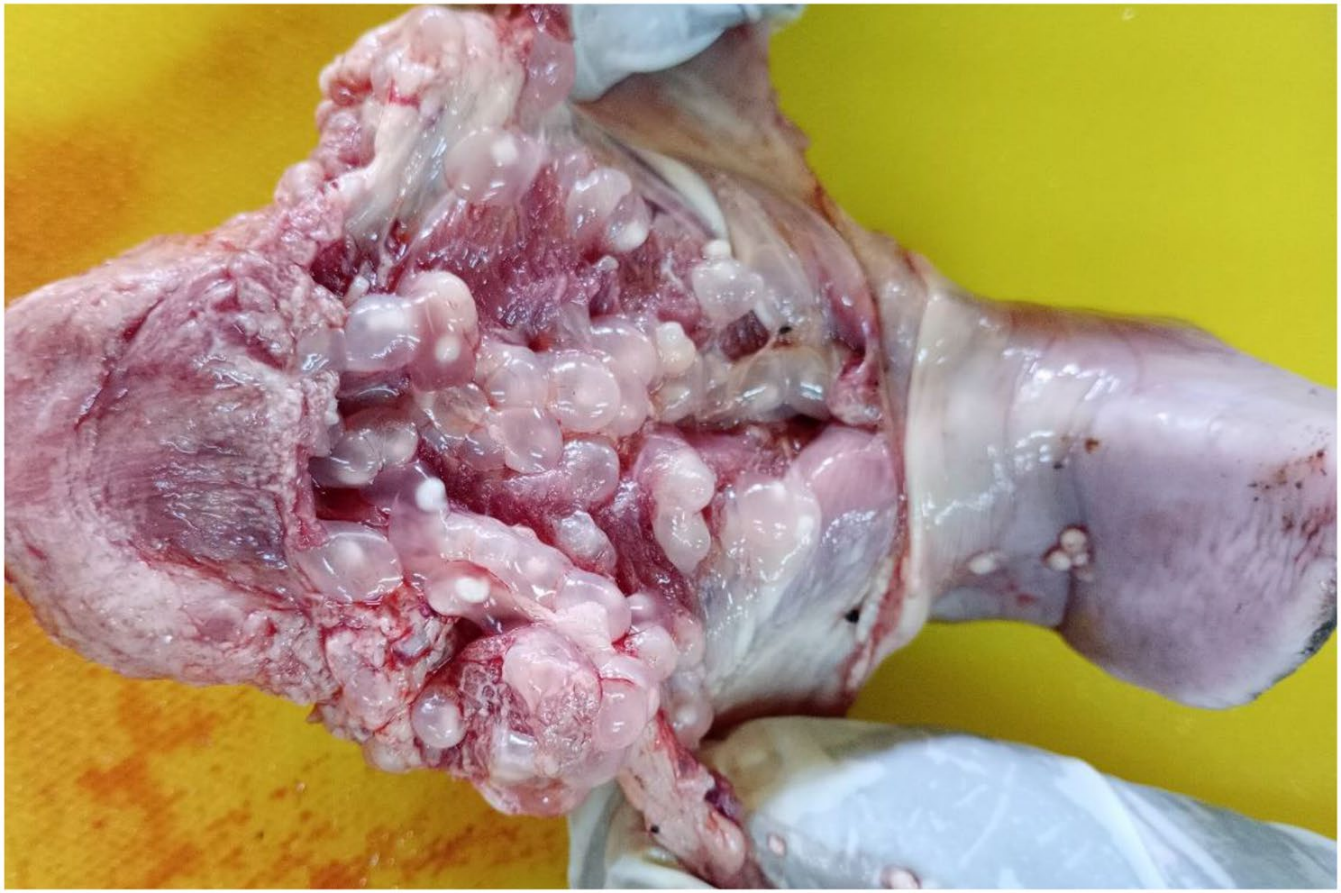
An example of a tongue heavily infected with *Taenia solium* cysticerci

By meat inspection, 14 out of 576 pigs (2.4%, 95% CI: 1.3-4.0) were positive while by partial carcass dissection 67 out of 576 pigs (11.6%, 95% CI: 9.1–14.5) were positive for *T. solium* cysticercosis, respectively (Table 1). Of the 67 pigs positive by partial carcass dissection, 66 (98.5%) were confirmed molecularly, leading to a prevalence based on molecular confirmation of 11.5% (95% CI: 9-14.4), including 26 pigs (9.0%, 95% CI: 6–13) in Bujumbura city, 24 pigs (16.7%, 95% CI: 11-23.8) in Kayanza province and 16 pigs (11.1%, 95% CI: 6.5–17.4) in Ngozi province. Significant differences in prevalence were observed in pig breed and origin (Table 1). The cysticerci intensity in the dissected organs and muscles of pigs infected with *T. solium* ranged from 1 to 1449 cysticerci, with an average of 80 cysticerci. Most cysticerci (76.1%) were found and counted in the masseter muscles, followed by the tongue (18.8%) and the heart (5.1%). The number of masseter muscle cysticerci was significantly higher than in the tongue and heart (*p* < 0.05) (Table 2). In organs and muscles, the majority of cysticerci were viable (88.3%) (Figs. 3, 4 and 5). Six-point-four percent (6.4%) were calcified cysticerci and 5.3% degenerated. No significant differences were observed in cysticerci intensity by province, sex, age and breed (Table 3). Based on infection intensities, 46 pigs (68.7%) infected with *T. solium* had light infections, 9 pigs (13.4%) had moderate infections, and 12 pigs (17.9%) had heavy infections (Table 4).

**Fig. 6:**
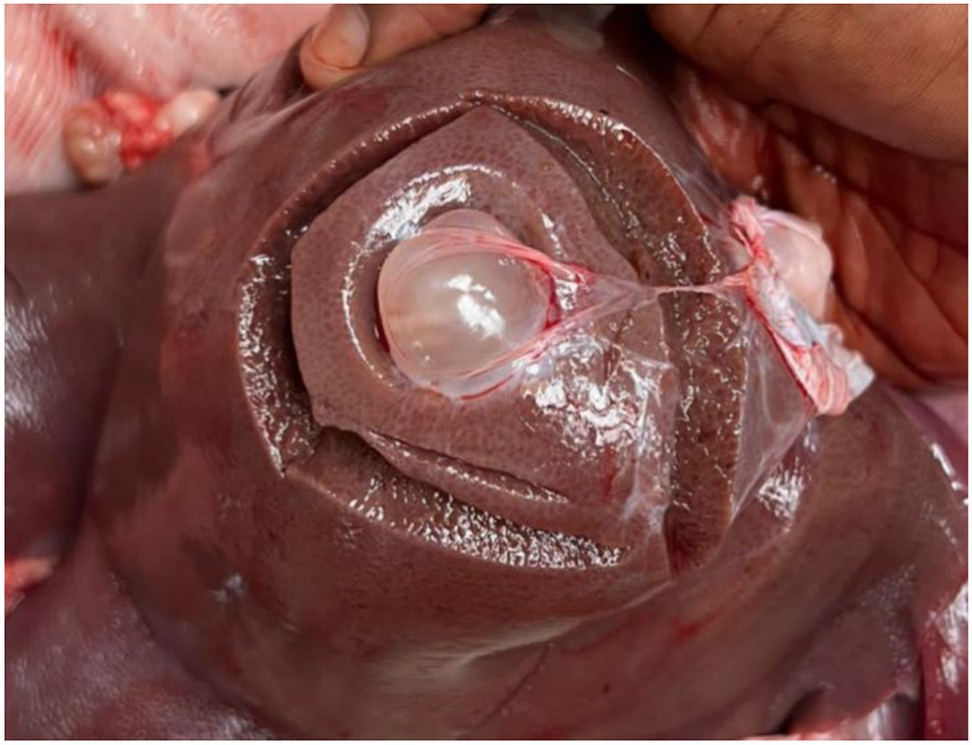
Liver infected with *Taenia hydatigena* cysticerci

**Fig. 7:**
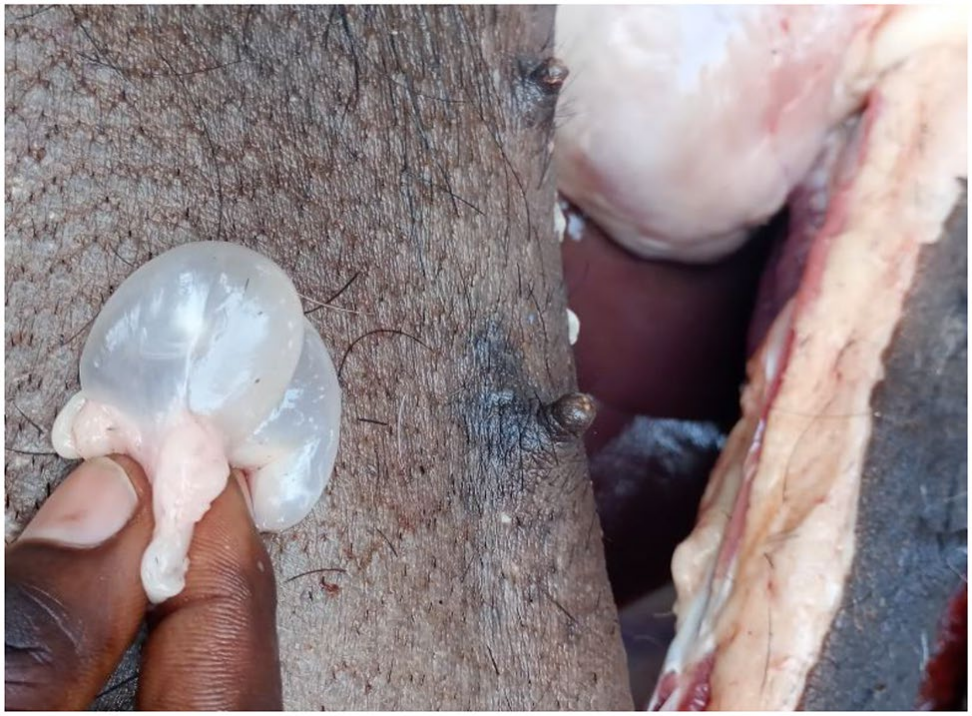
Large *Taenia hydatigena* cysticerci found in the abdominal cavity (mesentery)

**Fig. 8:**
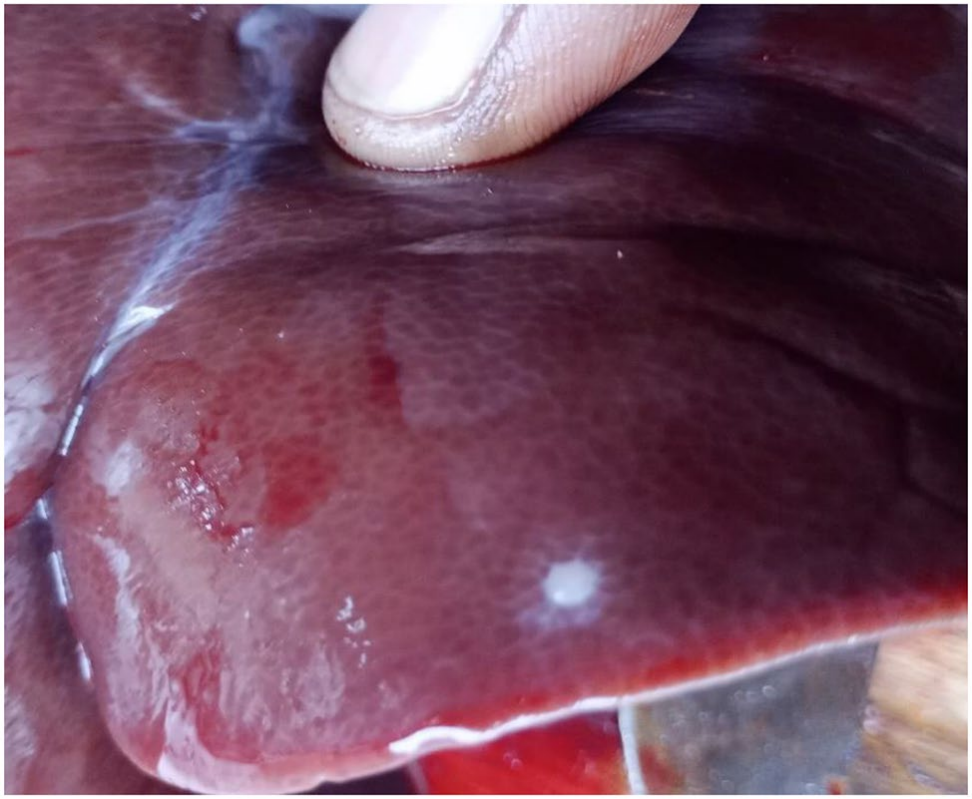
Liver with a suspected lesion (small white nodule)

Regarding *T. hydatigena* infection, 32 out of 576 pigs (5.5%, 95% CI: 3.8–7.8) were suspected of being infected during the inspection, including 7 pigs (1.2%, 95% CI: 0.3–2.1) having large *T. hydatigena* cysticerci in the liver and mesentery and 25 pigs (4.3%, 95% CI: 2.8–6.3) with small cysticerci or lesions in the liver (Figs. 6, 7 and 8). Of those 32 pigs suspected of *T. hydatigena* infection, 24 (7 with large cysticerci in the liver and mesentery and 17 with small cysticerci or lesions in the liver) were confirmed molecularly to be positive for *T. hydatigena*, and one with a small cysticercus in the liver was confirmed molecularly to be positive for *T. solium*. The overall prevalence of *T. hydatigena* infection was 4.2% (95% CI: 2.7–6.1), including 12 pigs (4.2%, 95% CI: 2.2–7.2) in Bujumbura city, 6 pigs (4.2%, 95% CI: 1.5–8.9) in Kayanza province and 6 pigs (4.2%, 95% CI: 1.5–8.9) in Ngozi province. The pig with the small cysticercus in the liver, which was confirmed positive for *T. solium* cysticercosis, also tested positive during both partial carcass dissection and molecular analyses. In addition, 2 pigs were co-infected with *T. solium* and *T. hydatigena*. The first pig had *T. solium* cysticerci in the masseter muscles and *T. hydatigena* cysticerci in the mesentery. The second pig had *T. solium* cysticerci on the tongue and *T. hydatigena* cysticerci in the liver. Seven negative samples at PCR-RFLP from liver suspected lesions were also negative for *Echinococcus* spp. and *Sarcocystis spp.* using multiplex PCR.

**Table 1:** Distribution of the prevalence of *Taenia solium* cysticercosis by meat inspection and partial carcass dissection

Variables	*N*	*P*_MI_	MI % (95% CI)	*P*_PCD_	PCD % (95% CI)	χ^2^_PCD_	*p*-value
Provinces							
Bujumbura city	288	5	1.7 (0.6-4)	26	9.0 (6–13)	5.46	0.065
Kayanza	144	7	4.9 (2-9.8)	24	16.7 (11-23.8)		
Ngozi	144	2	1.4 (0.2–4.9)	17	11.8 (7.0-18.2)		
Slaughter slabs							
National slaughterhouse	144	1	0.7 (0.0-3.8)	14	9.7 (5.4–15.8)	5.93	0.431
Gikoma	144	4	2.8 (0.8-7)	12	8.3 (4.4–14.1)		
Kayanza	104	6	5.8 (2.2–12.1)	18	17.3 (10.6–26)		
Muhanga	40	1	2.5 (0.1–13.2)	6	15 (5.7–29.8)		
Ngozi	79	1	1.3 (0.0-6.9)	10	12.7 (6.2–22.1)		
Busiga	34	1	2.9 (0.1–15.3)	4	11.8 (3.3–27.5)		
Gashikanwa	31	0	0.0	3	9.7 (2-25.8)		
Sex							
Male	256	4	1.6 (0.4-4)	23	9 (5.8–13.2)	2.69	0.101
Female	320	10	3.1 (1.5–5.7)	44	13.8 (10.2–18)		
Age							
6–12 months	285	4	1.4 (0.4–3.6)	29	10.2 (6.9–14.3)	0.90	0.343
≥ 13 months	291	10	3.4 (1.7–6.2)	38	13.1 (9.4–17.5)		
Breed							
Local	228	9	4 (1.8–7.4)	39	17.1 (12.5–22.6)	10.14	0.001*
Crossed	348	5	1.4 (0.5–3.3)	28	8.1 (5.4–11.4)		
Origin of pigs							
Bujumbura	55	0	0.0	4	7.3 (2-17.6)	11.54	0.042*
Gitega	15	1	6.7 (0.2–32)	4	26.7 (7.8–55.1)		
Karusi	67	0	0.0	2	3 (0.4–10.4)		
Kayanza	221	8	3.6 (1.6-7.0)	31	14.0 (9.7–19.3)		
Kirundo	28	3	10.7 (2.3–28.2)	5	17.9 (6.1–36.9)		
Ngozi	190	2	1.1 (0.1–3.8)	21	11.1 (7-16.4)		
Total	576	14	2.4 (1.3-4.0)	67	11.6 (9.1–14.5)	-	-

N: Number of examined pigs, P: Number of infected pigs, MI: Meat inspection, PCD: Partial carcass dissection, CI: confidence interval, χ^2^: chi-square, *significant

**Table 2:** *Taenia solium* cysticerci intensity and stages in organs and muscles

Organs and muscles	Infected pigs (%)	Viable	Degenerated	Calcified	Total	%	*p*-value
Heart	22 (32.8)	205	2	64	271	5.1	< 0.001 (Ref)
Tongue	39 (58.2)	891	28	82	1001	18.8	0.091
Masseter	40 (59.7)	3610	254	194	4058	76.1	< 0.001*
Total	-	4706 (88.3%)	284 (5.3%)	340 (6.4%)	5330	100	
Only tongue	20 (29.8)	24	0	5	29	0.6	
Only heart	6 (9)	2	0	5	7	0.1	
Only masseter	20 (29.8)	100	5	8	113	2.1	
Tongue + heart	1 (1.5)	0	0	5	5	0.1	
Tongue + masseter	5 (7.5)	591	228	53	872	16.4	
Heart + masseter	2 (3)	23	0	5	28	0.5	
Tongue + heart + masseter	13 (19.4)	3966	51	259	4276	80.2	
Total	67 (100)	4706 (88.3%)	284 (5.3%)	340 (6.4%)	5330	100	

*Significant

**Table 3:** *Taenia solium* cysticerci intensity in infected pigs by province, sex, age and breed

Variables	Infected pigs	Total number and mean of cysticerci per infected pig	% of all cysticerci	*p*-value
Provinces				
Bujumbura city	26	1738 (66.8)	32.6	< 0.001 (Ref)
Kayanza	24	2809 (117)	52.7	0.317
Ngozi	17	783 (46.1)	14.7	0.546
Sex				
Female	44	3052 (69.4)	57.3	< 0.001 (Ref)
Male	23	2278 (99)	42.7	0.487
Age				
6–12 months	29	1353 (46.7)	25.4	< 0.001(Ref)
≥ 13 months	38	3977 (104.7)	74.6	0.097
Breed				
Crossed	28	1708 (61)	32.0	< 0.001 (Ref)
Local	39	3622 (92.9)	68	0.394

**Table 4:** Proportion of *Taenia solium* infection levels in infected pigs per province

Infection intensity level	Bujumbura city	Kayanza	Ngozi	Total	%	Mean of cysticerci per infected pig
Light (1–10 cysticerci)	19	13	14	46	68.7	2
Moderate (11–100 cysticerci)	3	6	0	9	13.4	49
Heavy (101–1449 cysticerci)	4	5	3	12	17.9	402
Total	26	24	17	67	100	80
Light (diagnosed by MI)	0	0	0	0/46	0.00	-
Moderate (diagnosed by MI)	1	3	0	4/9	44.4	-
Heavy (diagnosed by MI)	4	4	2	10/12	83.3	-
Total	5	7	2	14/67	20.9	-

MI: Meat inspection

### Prevalence of *T. gondii* infection in pigs based on serological analyses

Of the 576 pig sera, antibodies against *T. gondii* were detected in 102 pigs (17.7%, 95% CI: 14.7–21.1) (Table 5). *Toxoplasma gondii* seropositivity was significantly associated with sex, location of slaughter slabs, and origin of pigs (Table 5).

**Table 5:** Distribution of the prevalence of toxoplasmosis in pigs in Burundi using indirect ELISA

Variables	Examined pigs	Infected pigs	Prevalence % (95% CI)	Chi-square	*p*-value
Provinces					
Bujumbura city	288	52	18.1 (13.9–23)	0.43	0.807
Kayanza	144	23	16 (10.4–23)		
Ngozi	144	27	18.8 (12.7–26.1)		
Slaughter slabs					
National slaughterhouse	144	17	11.8 (7.0-18.2)	16.45	0.012*
Gikoma	144	35	24.3 (17.6–32.2)		
Kayanza	104	15	14.4 (8.3–22.7)		
Muhanga	40	8	20 (9.1–35.7)		
Ngozi	79	21	26.6 (17.3–37.7)		
Busiga	34	4	11.8 (3.3–27.5)		
Gashikanwa	31	2	6.5 (0.8–21.4)		
Sex					
Male	256	35	13.7 (9.7–18.5)	4.67	0.031*
Female	320	67	20.9 (16.6–25.8)		
Age					
6–12 months	285	48	16.8 (12.7–21.7)	0.18	0.667
≥ 13 months	291	54	18.6 (14.3–23.5)		
Breed					
Local	228	44	19.3 (14.4–25.0)	0.49	0.486
Crossed	348	58	16.7 (12.9–21.0)		
Origin of pigs					
Bujumbura	55	20	36.4 (23.8–50.4)	23.76	< 0.001*
Gitega	15	1	6.7 (0.2–32)		
Karusi	67	4	6 (1.7–14.6)		
Kayanza	221	32	14.5 (10.1–19.8)		
Kirundo	28	5	17.9 (6.1–36.9)		
Ngozi	190	40	21.1 (15.5–27.5)		
Total	576	102	17.7 (14.7–21.1)	-	-

CI: Confidence interval, *significant

## Discussion

This study is the first to report the occurrence of *T. hydatigena* and *T. gondii* in pigs in Burundi. The prevalence of *T. solium* cysticercosis by partial carcass dissection was almost five times higher than that detected by meat inspection. This could be explained by the fact that partial carcass dissection is more sensitive (81%) compared to meat inspection (22.1%), particularly for light infections [23, 24], which represented the majority in our study (68.7%). Meat inspection, the routinely implemented technique in Burundi, detected only heavy and some moderate infections and failed to detect all light infections, which could lead to significant public health problems due to the consumption of these infected carcasses, especially as most detected cysts were viable. In this study, the prevalence based on meat inspection was low. Similar low prevalences were reported in Kenya (1.8%), India (1.4%), Uganda (0.6%), Madagascar (4.6%) and South Africa (5%) [25–29]. This low prevalence in Burundi could be due to tongue palpation carried out before the sale or slaughter of pigs, which led to the clandestine and home slaughter of infected pigs. This is a common practice in Burundi to avoid carcass condemnation in slaughter slabs [19]. In a study conducted on pig farms in Burundi, a high prevalence of *T. solium* cysticercosis (15.5%) in pigs based on tongue palpation was reported [19]. Despite the low sensitivity of tongue palpation in light infections (21%) [24], it is still a rapid and cheap diagnostic tool used by pig traders and butchers in Burundi to ensure they bring healthy pigs to the slaughter slabs. However, this practice can lead to huge losses for farmers when a pig is found infected with cysticerci on the tongue, as the selling price is discounted up to 80% for an infected pig [19, 30]. This can have significant implications for public health because infected pigs do not reach slaughter slabs for meat inspection [19]. They are either slaughtered at home for family and neighbourhood consumption or they undergo clandestine slaughter by butchers for public sale at various outlets, thus posing a high risk for *T. solium* taeniasis. This practice of consuming infected pork from clandestine or home slaughter ensures sustained local transmission, as often pig farmers will get infected with pork tapeworms, resulting in greater environmental contamination with *Taenia* eggs and an increased risk of pig infection within the communities.

Partial carcass dissection, an alternative technique to full carcass dissection (the gold standard), was used to estimate *T. solium* cysticerci intensities and stages in organs and muscles [23]. Similar prevalences based on partial carcass dissection including tongue, heart, and masseter muscles were estimated at 12.1% in Peru and 17.6% in Cameroon [23]. With a sensitivity of 81%, partial carcass dissection is probably the better technique under research conditions, due to cost savings in purchasing pigs when the sample size is large, as well as the reduced labour and time required to dissect the entire carcass [22, 23]. Although this technique is more sensitive than meat inspection, implementing this technique routinely at pig slaughter slabs is impossible due to its laborious and time-consuming nature, coupled with the loss of value in dissected meat and potential unfamiliarity among meat inspectors.

In this study, local breed pigs were more infected than cross breeds and pigs from Gitega and Kirundo were more infected than those from other provinces. This could probably be explained by the fact that cross breeds were mainly found on large farms with improved husbandry practices, regular deworming schedules, and commercial feeds, in contrast to black pigs kept on small-scale farms with traditional farming practices, including free-ranging. Furthermore, in the densely populated provinces of Burundi (Gitega, Kirundo, Kayanza, Muyinga, and Ngozi), pigs are typically raised on small-scale farms using traditional methods intended to generate household income with minimal inputs [19, 31]. This approach may increase the risk of exposing pigs in these areas to *T. solium* eggs. Based on infection intensity, 65.4% of pigs infected with cysticerci in Tanzania and 76% in Zambia had light to moderate infections, consistent with our results [22, 32]. It was demonstrated that free-range pigs were likely to have cysticercosis with moderate or heavy infections due to the ingestion of human faeces released during open defecation, which might contain large quantities of pork tapeworm eggs [33]. In addition, light infections in pigs could result from a low dose of pork tapeworm eggs, potentially ingested by roaming in the field, herbs brought into the pig pens, or consuming contaminated water [33]. All these factors could explain the three levels of cysticercosis infection in Burundi associated with pig farming systems, including pigs raised in pens, partially penned pigs and free-range pigs [19]. Findings in Cameroon corroborated the heavier infections in masseter muscles compared to the tongue and heart [34]. Given the high *T. solium* cysticerci intensities in masseter muscles and the tongue, the Ministry in charge of livestock must enforce the inspection of these muscles, which are currently absent from the meat inspection policy [35]. In this study, all pigs with light and moderate infections and most pigs with heavy infections entered the food chain. This could be attributed to a loophole in the meat inspection policy, which allows pigs with light and moderate infections to be delivered for human consumption [35]. Additionally, a lack of vigilance among meat inspectors during the inspection period was noted, which could lead to infected carcasses reaching pork sales outlets. Thus, rigorous meat inspection and good decisions at the slaughter slabs need to be made to prevent moderately and heavily infected pork from reaching consumers. Moreover, it is crucial to carefully manage pig carcasses with light infections that are not detected during routine meat inspections. These pig carcasses could pose a potential risk for taeniasis as they appear safe, leading consumers to underestimate the risk [33]. Hence awareness of culinary and consumption practices to limit the transmission of the parasite should be raised. Moreover, education campaigns on improved pig husbandry practices, hygiene, sanitation, and treatment of pork tapeworm carriers would be important for preventing *T. solium* cysticercosis contamination in pigs and humans [36].

Molecular analyses confirmed the presence of the non-zoonotic tapeworm *T. hydatigena* with a prevalence of 4.2% in Burundi, which is consistent with the prevalence reported in Africa [37]. The presence of this parasite in pigs in Burundi could significantly indicate the presence of tapeworm in dogs, which could get infected by visiting slaughter slabs, consuming entrails, and subsequently contaminating the environment with their faeces [9]. In agreement with this study, co-infections with *T. hydatigena* and *T. solium* were also found in Tanzania, Cameroon, and Zambia [22, 34, 38]. Considering that dogs could have access to all slaughter slabs or home slaughters, proper management of offal that may contain *T. hydatigena* cysticerci, combined with improved pig husbandry practices, should be implemented to control the transmission of this parasite in Burundi.

This study revealed the presence of *T. gondii* in pigs in Burundi. The findings of this study corresponded to the seroprevalence in pigs reported in Africa, which ranges from 17 to 34%, with an average of 25% [39]. Higher seropositivity was observed in Kenya (34.5%), Ethiopia (32.1%), South Africa (33.6%), and Ghana (39%) [40–43]. In addition, similar seropositivity figures were found in South America and Asia, but they were higher than those in Europe [39]. This difference in seropositivity between countries could be associated with variations in pig management systems, climatic conditions (temperature, rainfall, humidity), hygienic conditions, and the density of cats and rodents on pig farms [10]. Although this indirect ELISA for detecting antibodies against the *T. gondii* P30 protein in serum demonstrates better sensitivity and specificity [44, 45], it does not imply that all seropositive pigs had viable *T. gondii* cysts, as it detects antibodies and cannot distinguish between past and active infections. No cross-reactions with other apicomplexan parasites have yet been reported in pigs using this ELISA kit [44, 45]. It was shown that pigs raised in extensive systems had a higher probability of ingesting sporulated oocysts during free-roaming, as well as animal tissues from birds or rodents and even contaminated water, in contrast to pigs raised in intensive systems [10, 46]. In Burundi, cats are usually kept to control rodents within households, and their access to pig housing, feed, and water can explain the transmission of the parasite to penned pigs. A study in the United States of America showed that a prevalence of less than 1% was observed in confined pigs without access to cats and rodents, highlighting the need to strengthen control methods to prevent their introduction to pig farms [47]. As a zoonotic parasite, this seropositivity in Burundi indicated that pork consumers could be highly exposed to this parasite via undercooked pork consumption. There is no way to prevent infected carcasses from reaching the food chain, as routine meat inspections cannot detect *T. gondii* cysts in meat tissues [48]. Pork consumers need to ensure that pork is well cooked, fried, or roasted to avoid contamination by this parasite. These good practices could also be applied to *T. solium* cysticercosis to help reduce the risk of human infection. Cooking meat at 67 °C for more than 3 min was recommended as an effective way to kill *T. gondii* cysts [49]. In addition, community awareness about pig management practices, cat keeping, pork preparation and consumption, hygiene, and sanitation should be implemented to prevent animal and human toxoplasmosis infections [50].

## Conclusions

The study’s findings indicate that two foodborne zoonotic parasites, *T. solium* and *T. gondii*, are endemic in Burundi. These results show evidence of potential public health risks for the local population due to the consumption of pig carcasses infected with these parasites. The prevalence of *T. solium* cysticercosis in this study could probably be underestimated because pigs found to be infected with cysticerci during tongue palpation in pig farms could not reach official slaughter slabs and the sensitivity of partial carcass dissection is not 100%. Effective control strategies should be implemented to avoid the perpetual contamination of pigs and humans with these zoonotic parasites. Possible control strategies to be considered tackling both parasites are improved pig farming practices, better hygiene and sanitation, increased meat inspection and monitoring of infected pigs, risk-free culinary practices, and treatment of tapeworm carriers.

## Methods

### Study area

**Fig. 9:**
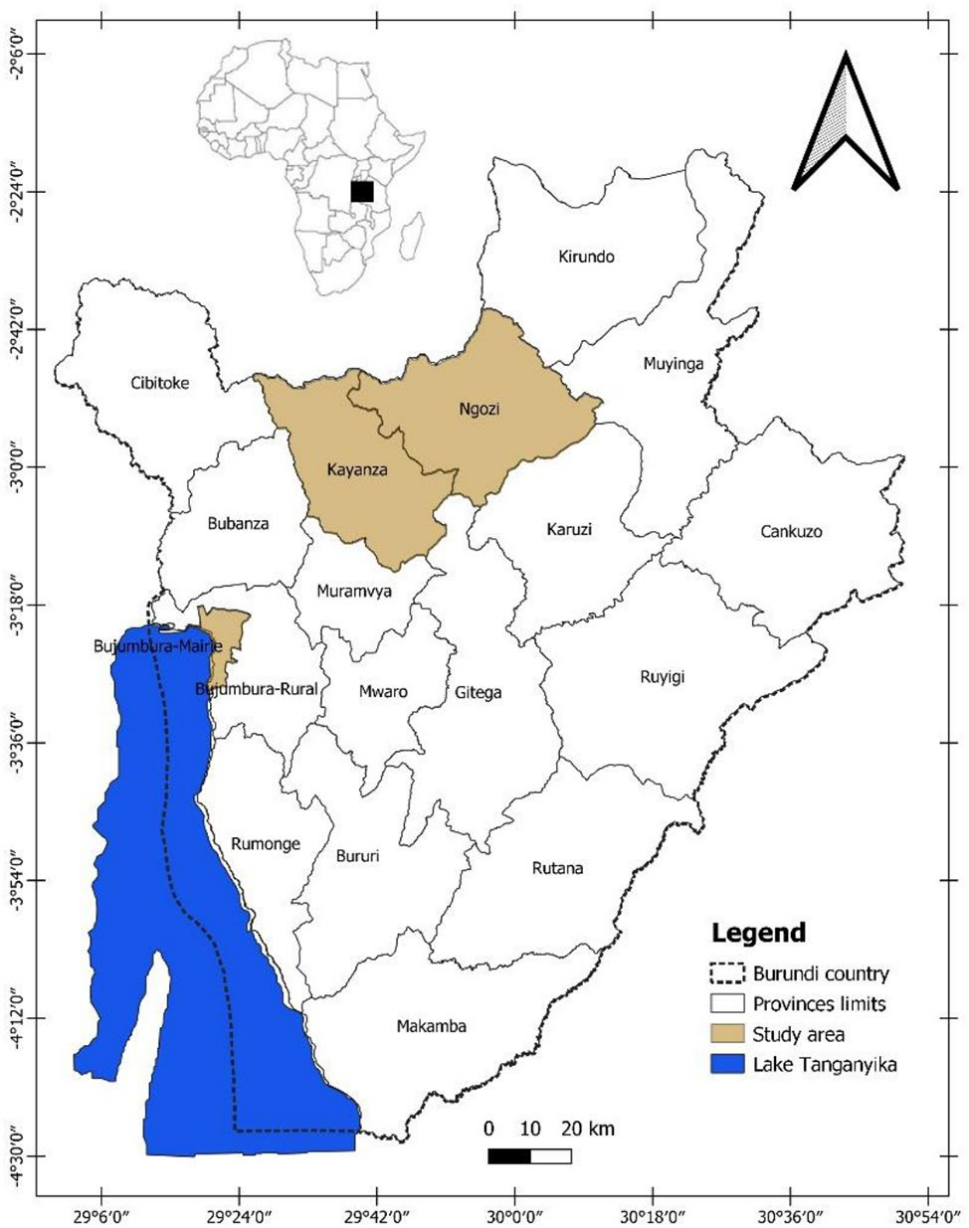
Map of Burundi showing the study area

The study was conducted in slaughter slabs located in urban (Bujumbura city, economic capital) and rural areas (Ngozi and Kayanza provinces) of Burundi (Fig. 9). Bujumbura city was chosen due to the high number of pigs from the countryside that are slaughtered and marketed in the city. Kayanza and Ngozi provinces were selected because they are densely populated, and extensive pig farming systems are a major source of income for pig farmers [51].

### Study design and sample size

A cross-sectional study including field and laboratory work was conducted from October 2023 to March 2024. The number of pigs slaughtered per day was an inclusion criterion when selecting slaughter slabs for the study. Thus, the national slaughterhouse of Bujumbura, the slaughter slab of Gikoma in Bujumbura city; slaughter slabs located in Kayanza and Muhanga communes in Kayanza province; and slaughter slabs located in Ngozi, Gashikanwa and Busiga communes in Ngozi province were included in this study. When more than 10 pigs were available for slaughter per day, a random sampling of pigs was applied. If less than 10 pigs were available, all the available pigs meeting the inclusion criteria were selected. Pigs were selected according to their sex, age, breed, and origin. Only apparently healthy pigs older than 5 months were included in the study.

The sample size of pigs was determined using the formula N = Z^2^pq/L^2^ [52]. A prevalence of porcine cysticercosis estimated at 15.5% by tongue palpation in Burundi [19] and a seroprevalence of toxoplasmosis in pigs estimated at 25% in Africa [39] were considered to calculate the required sample size. Since porcine cysticercosis and toxoplasmosis were assessed on the same pigs at the same slaughter slabs, a sample size of 288 pigs was considered. Considering both urban and rural areas, a total of 576 pigs were sampled, including 288 pigs from Bujumbura city and 288 pigs from Kayanza and Ngozi provinces.

### Field data collection

Pigs of pig traders and butchers who agreed to offer their pigs for the study were included. Information on the age, sex, breed, and origin of each pig was recorded. Upon pig slaughter, jugular vein blood samples were collected into 50 mL Falcon tubes. After pig slaughter, the meat inspector (veterinarian) conducted the meat inspection according to the procedures in Burundi [35]. Meat inspection regulations for *T. solium* cysticercosis in Burundi include incising of thigh muscles, abdominal wall, psoas, diaphragm, intercostal muscles, larynx and heart [35]. In the slaughter slabs, pig carcasses were sometimes inspected by making a deep incision with a knife in the muscles of the fore and hind limbs, as well as the heart, to search for *T. solium* cysticerci. In the national slaughterhouse of Bujumbura, the deep incision of the forelimb muscles, heart and tongue was conducted on each pig carcass for searching *T. solium* cysticerci. In addition, during the pig carcass inspection the pigs’ abdominal cavity, particularly the liver and mesentery, was inspected for *T. hydatigena* cysticerci. *Taenia hydatigena* cysticerci were identified macroscopically if they were moderately large (≥ 2 cm), translucent, filled with clear fluid, and had a visible white spot indicating a long-necked scolex [53]. Small cysticerci and other unclear lesions in the liver showing cysticerci stages were considered suspected lesions for either *T. hydatigena* or *T. solium.* Large *T. hydatigena* cysticerci found in the liver and mesentery were collected separately in 50 mL Falcon tubes containing 70% ethanol, while suspected liver lesions were collected separately in 2 mL cryovials containing 70% ethanol for further molecular analysis. After meat inspection, the tongue, heart, and masseter muscles from each inspected pig were purchased from butchers and pig traders and packaged in small bags. The blood and meat samples were labelled and placed in cool boxes and transported to the National Veterinary Laboratory of Bujumbura for serological analysis and partial carcass dissection. Samples for molecular analysis were sent to the laboratory of the Institute of Tropical Medicine in Antwerp, Belgium and the Laboratory of Foodborne Parasitic Zoonoses at Ghent University, Belgium for further analyses. All the results from the meat, partial carcass dissection and molecular analysis were recorded.

## Laboratory analyses

### Partial carcass dissection

The tongue, heart, and masseter muscles were dissected by making slices less than 5 mm thick and inspecting for *T. solium* cysticerci [54]. The cysticerci were counted and classified as viable, degenerated and calcified based on their macroscopic appearance. The cysticerci were classified as viable if they had a translucent fluid with visible whitish protoscolices; degenerated if they had damaged viscous walls or absence of cystic fluid; and calcified if they had solid caseous masses [22]. Infection intensities were estimated based on the number of cysticerci: light (1–10 cysticerci), moderate (11–100 cysticerci), and heavy (> 100 cysticerci). A maximum of 5 cysticerci from the collected heart, tongue and masseter muscles from each infected pig carcass were collected and stored together in 2 mL cryovials with 70% ethanol for molecular analysis.

### Serological analysis

The blood tubes were allowed to clot overnight at 4 °C to obtain serum. The serum was aliquoted, transferred to 2 ml cryovials, and stored at -20 °C until analysis. Serum samples were tested by an indirect ELISA for the detection of antibodies against the *T. gondii* P30 protein in serum, plasma, and meat juice from multiple species (ID Screen^®^ Toxoplasmosis Indirect Multi-species). This ELISA was performed following the manufacturer’s recommendations.

### Molecular analysis

All collected cysticerci and suspected lesions were sent to and analysed in the Helminthology Laboratory of the Institute of Tropical Medicine in Antwerp, Belgium. The polymerase chain reaction-restriction fragment length polymorphism (PCR-RFLP) to identify and differentiate *Taenia* species was adopted. Genomic DNA from all samples was extracted using the DNeasyBlood and Tissue Extraction kit according to the manufacturer’s instructions (QIAGEN, Hilden, Germany). PCR was used to amplify a mitochondrial 12S rDNA gene fragment with the primer set ITM TnR-TaenF and nTAE [55]. Subsequently, RFLP was used to differentiate *Taenia* spp. using restriction enzymes, including DdeI, HinfI, and HpaI [55, 56]. Therefore, the PCR-RFLP results were interpreted by analysing the band sizes specific to *T. solium* and *T. hydatigena*. Negative genomic DNA samples from liver suspected lesions were then tested with the multiplex PCR [57] for *Echinococcus* spp. and *Sarcocystis* spp. in the Laboratory of Foodborne Parasitic Zoonoses at Ghent University, Belgium.

### Data analysis

Data from the field and laboratory analyses were entered in Microsoft Excel and then exported to R Software, version 4.3.3 [58]. Descriptive statistical analyses including frequencies, means, proportions, and 95% confidence intervals (CI) were performed. Associations between disease prevalence and province, age, sex, breed, and origin of pigs were estimated using the Chi-square test. In addition, Poisson regression and negative binomial regression models were used to assess the significance of cysticerci intensity counted in the heart, tongue and masseter muscles and the effect of age, sex, breed, and province on the cysticerci intensity in organs/muscles. The variable was statistically significant when the p-value was less than 0.05.

## Data Availability

The data supporting the conclusions of this article are included within the article. The raw datasets are available from the corresponding author upon reasonable request.

## Abbreviations

CI: Confidence interval
ELISA: enzyme-linked immunosorbent assay
MI: Meat inspection
PCD: Partial carcass dissection
PCR: RFLP-Polymerase chain reaction-restriction fragment length polymorphism
